# P53-mediated in vitro inhibition of PhIP-induced oxidative damage by myricetin bulk and nano forms in healthy lymphocytes

**DOI:** 10.1007/s00204-021-03010-6

**Published:** 2021-02-24

**Authors:** Shabana Akhtar, Diana Anderson, Talha Muhammad Azam, Arif Malik

**Affiliations:** 1 Institute of Molecular Biology and Biotechnology (IMBB) and Centre for Research in Molecular Medicine (CRiMM), University of Lahore, Defence road campus, Lahore, Pakistan; 2 School of Chemistry and Biosciences, University of Bradford, Bradford, BD7-1DP UK

## Introduction

We conducted this small study to follow on our previous research (Akhtar et al. 2020a) where we have shown that myricetin bulk (MYR B) and nano (MYR N) forms can potentially suppress 2-Amino-1-methyl-6-phenylimidazo[4,5-b]pyridine (PhIP)-induced DNA damage in lymphocytes from pre-cancerous patients and those from healthy individuals. We analysed the effects of PhIP treatment on the induction of DNA damage, strand breaks formation, kinase ataxia telangiectasia and Rad3-related protein (ATR) regulation, and p53 m RNA expression levels. We showed that PhIP causes a significant level of oxidative DNA damage in lymphocytes from both investigative groups assessed using the comet and micronucleus assays. Moreover, we have shown the modulating effects of MYR B and MYR N on PhIP-induced metabolic changes of these factors.

In the current study, we further investigated the molecular mechanisms involved in the protective and anti-mutagenic effects of myricetin against PhIP-induced damage in peripheral lymphocytes from healthy individuals. We determined the anti-oxidant potential of myricetin against PhIP-induced oxidative stress by analysing the levels of intracellular anti-oxidant enzyme, glutathione (GSH). Also we studied the influence of these compounds on P53 and Bcl-2 protein levels in isolated lymphocytes using the Western blotting technique which is an enhancement and was not addressed in our novel study.

The current project involving the use of human peripheral lymphocytes has been granted ethical approval by Leeds East Ethics Committee (IRAS Reference No.:12/YH/0464) and the University of Bradford’s Sub-Committee for Ethics in Research involving healthy Human Subjects (Reference No.: 0405/8). Peripheral blood samples were collected through venepuncture from three healthy individuals after obtaining informed consent. The research support and governance office of Bradford Teaching Hospitals NHS Foundation also agreed the research (REDA number 1202).

Refer to our previous data (Akhtar et al. 2020a,c) for cell culture, reagents, cell survival, dose–response studies and Western blot technique (Akhtar et al. 2020b). However, the assay of cellular enzyme used in the present project is described below.

Briefly, for the cellular enzyme assay (All step to be carried out in the fume hood and on ice) isolated lymphocytes (10,000–100,000/well), supplemented with complete medium (RPMI medium along with 15% foetal bovine serum (FBS) and 1% penicillin streptomycin) (All chemicals, Invitrogen, UK) were harvested overnight in 6-well plates. The following day the cells were treated with chemicals for 1 h. Then the medium was carefully removed and the cells were washed with cold phosphate buffer saline (PBS) and re-suspended in 100 µl of cold lysis buffer supplemented with 10 µl of protease inhibitors (both from Fisher Scientific, UK). Cells were thoroughly mixed by pipetting and centrifuged at 400*g* for 5 min to remove the supernatant. The supernatant (sample) was collected and kept on ice for further use. The assay was further performed according to the manufacturer’s protocol using GSH/GSSG Ratio detection assay kit (fluorometric green) (Abcam, UK). Fluorescence was measured at 490/520 nm using Promega Glumax explorer version 2.4.

Each experiment was repeated three times in different individuals. Results were expressed as means ± standard errors (SE). Graph Pad prism 9 was used to perform statistical analysis. The results were analysed using *t*-tests and two-way analysis of variance (ANOVA) to test differences between each treatment and control. A *p*-value of < 0.05 was considered statistically significant.

## Activity of intracellular anti-oxidant enzyme, GSH and change in GSH/GSSG ratio

It is believed that food mutagens damage the DNA by producing reactive oxygen species (ROS) and flavonoids act in an anti-oxidant manner to reduce this damage (Kurzawa-Zegota et al. 2012). Therefore, DNA damage caused by PhIP in lymphocytes from healthy individuals could possibly be because of dual mechanisms: CYP1A2-induced or ROS-induced genotoxicity. The findings from the current study confirm that myricetin is effectively able to prevent the DNA of lymphocytes from healthy individuals from PhIP induced-DNA damage. To determine whether the protective effects of myricetin are due to its anti-oxidant properties, we further investigated the effects of PhIP and co-supplementation of myricetin on the intracellular oxidative defence mechanisms by analysing the levels of anti-oxidant enzyme GSH. Glutathione is a major tissue anti-oxidant and the depletion of this crucial enzyme increases the susceptibility to oxidative stress, characterized by the accumulation of ROS (Gawryluk 2011). Glutathione is the smallest intracellular protein thiol molecule present in the cells that inhibits ROS (such as free radicals and peroxides) induced cell damage.

Glutathione is present in two forms, oxidised (GSSG) and reduced (GSH). In healthy cells, the total glutathione pool is mostly in the reduced form (GSH). It is the reduced one which protects against ROS by donating an equivalent ion to the one being detoxified. However, when cells are exposed to increased levels of oxidative stress, oxidized GSH (GSSG) starts accumulating and the ratio of GSSG to GSH rises. Hence, a bigger ratio of GSSG-to-GSH is an indication of oxidative stress (Roy and Sil 2012). Hydrogen peroxide is cleansed through GSH peroxidase (GPx), giving rise to GSSG which is then recycled back to the reduced form by GSH reductase (GR) (Meister 1988). Therefore, the conservation of sufficient GSH levels is crucial for protection against oxidative damage.

Since GSH is the main anti-oxidant defence system in our cells, a test was carried out to determine its intracellular levels by using a highly sensitive proprietary non-fluorescent dye which becomes fluorescent when reacted with GSH.

**Fig. 1:**
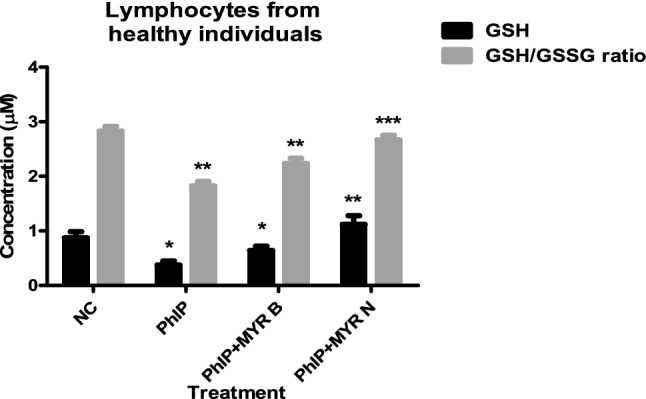
Effect of PhIP on GSH levels and GSH/GSSG ratios and modulation of PhIP-induced oxidative stress by MYR B and MYR N in healthy lymphocytes. Cells lysed to the concentration of 1 × 10^5^ cells/ml. Various treatment groups included the NC (negative control untreated), PhIP (100 µM) as a positive control (PC), MYR B (10 µM) + PhIP and MYR N (20 µM) + PhIP_._ PC was compared against the NC, however, other two treatment groups MYR B + PhIP and MYR N + PhIP were compared against the PC. (*) show the difference between the compared groups. *p < 0.01; **p < 0.01; ***p < 0.001

In-vitro treatment of lymphocytes from healthy individuals with mutagen PhIP (100 µM) alone and co-supplementation with MYR B (10 µM) and MYR N (20 µM) has shown a significant effect on GSH levels and GSH/GSSG ratio in healthy individuals. PhIP has significantly reduced GSH and GSH/GSSG ratios (*p* < 0.01) when compared to the untreated negative control. A reduction in GSH to GSSG ratio is a clear sign of oxidative stress which is evident from our results. However, MYR B and MYR N co-supplementation could effectively attenuate the adverse effects of PhIP and significantly retained the intracellular levels of GSH as well as increased the GSH/GSSG ratio (Fig. 1). The anti-oxidant, GSH activity was increased while oxidative stress was decreased enhancing the repairing capacity of the cells and positively affecting their proliferation (Maqbool et al. 2019). Increase in GSH levels after treatment with MYR B and MYR N co-supplementation supports the narrative that myricetin might be able to initiate DDR in the presence of oxidative stress caused by PhIP.

## Analysis of P53 and Bcl-2 expression in lymphocytes from healthy individuals

The results from our novel study proposed that myricetin may protect against the mutagenicity caused by PhIP in healthy lymphocytes by triggering the activation of ATR in P53-mediated DNA damage response (DDR) pathway (Akhtar et al. 2020a). Hence, contribute towards the survival path by the balancing of pro-survival and pro-death signals. To confirm this notion, we further investigated the effect of PhIP treatment and co-supplementation of myricetin bulk and nano on the post-transcriptional protein levels of P53 and the anti-apoptotic protein, Bcl-2 in healthy lymphocytes using Western blotting.

**Fig. 2:**
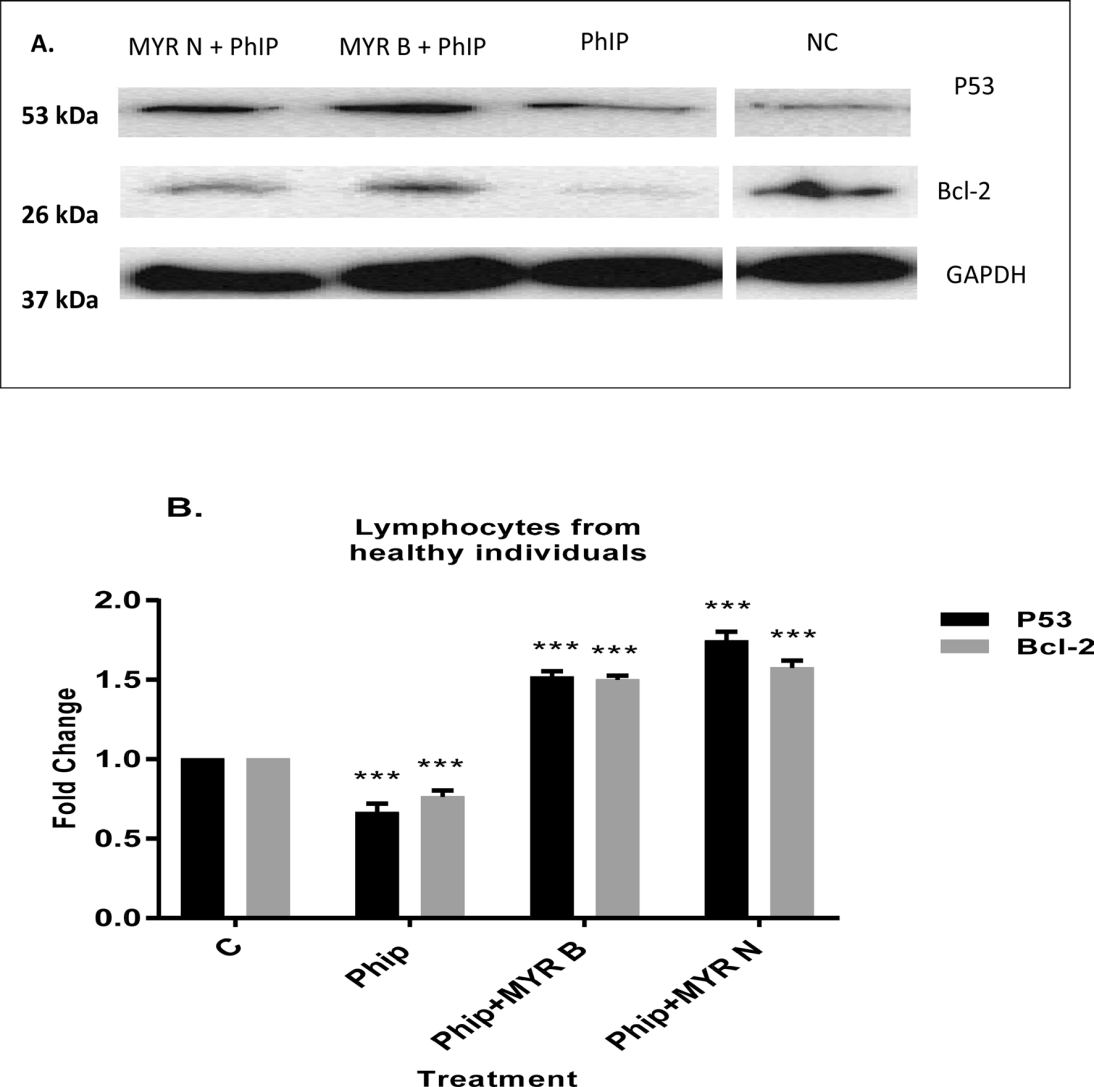
Modulating effects of myricetin bulk (MYR B) and nano (MYR N) on PhIP-triggered alterations in protein expression of P53 and Bcl-2 in healthy lymphocytes (**a**) Immunoblot analysis of the p53, and bcl-2 proteins in lymphocyte from healthy individuals treated with PhIP (100 µM), MYR B (10 µM) with PhIP and MYR N (20 µM) supplemented with PhIP. P53 and Bcl-2 expression was decreased after PhIP treatment. Supplementation of MYR B and MYR N significantly increased the expression levels of both proteins compared to PhIP alone treated group. GAPDH was used as an internal control protein to normalise the data. **b** Bar graphs exhibiting fold changes in protein expression levels. Data were represented as the mean ± SE of three experiments. ^***^P < 0.0001

The results demonstrated a significant down-regulation in the expression levels of both proteins upon PhIP treatment and significant attenuation of the PhIP-induced effects by myricetin supplementation. Figure 2 showed that P53 and Bcl-2 levels were decreased by 0.6-fold and 0.7-fold, respectively when healthy lymphocytes were exposed to PhIP (100 µM). However, a combination of PhIP with either MYR B or MYR N significantly increased the expression levels of both proteins. With MYR B administration, a 1.5-fold increase was observed in P53 and 1.49-fold increase in Bcl-2 expression levels. MYR N enhanced the levels of former to 1.7-fold and Bcl-2 was up-regulated by 1.52-fold. These results from Western blot analysis indicated that MYR B and MYR N might suppress the mutagenicity caused by PhIP in lymphocytes from healthy individuals by stimulating the expression levels of tumour-suppressor protein, P53 and anti-apoptotic protein, Bcl-2 ultimately causing a protective and anti-tumour effect. MYR N has shown more protective results compared to MYR B. We showed in our previous study that oxidative stress caused by PhIP triggered phosphorylation of kinase ATR which was further enhanced by myricetin bulk or nano treatment in healthy lymphocyte cells (Akhtar et al. 2020a). Tumour-suppressor, P53 gene was down-regulated by PhIP while both forms of myricetin caused significant conservation of the gene. Results were consistent with our present data on P53 protein regulation. As PhIP is believed to trigger cellular death while exhibits high levels of mutations in surviving cells (Gooderham et al. 2002). The up-regulation of kinase ATR and P53 protein upon myricetin co-supplementation with PhIP could be an indicative of a functional continuous repair mechanism. It has been shown that induction of oxidative stress triggers phosphorylation of P53, the formation of double strands breaks and suppression of anti-apoptotic signals ultimately leading to apoptosis (Shi et al. 2014; Cordani 2020). This only supports our PhIP-related results. However, our overall data suggest that myricetin bulk and nano forms might have suppressed the damage caused by PhIP in a P53 dependent manner promoting the anti-apoptotic protein, Bcl-2 and conferring the survival pathway.

To conclude our findings, MYR B and MYR N can potentially inhibit PhIP-induced oxidative stress by retaining the levels of intracellular anti-oxidant enzyme, GSH through a P53-regulated pathway.
